# Diverse grouping and mating strategies in the Critically Endangered Hainan gibbon (*Nomascus hainanus*)

**DOI:** 10.1007/s10329-022-00983-5

**Published:** 2022-03-24

**Authors:** Ping Li, Paul A. Garber, Yu Bi, Kun Jin, Xuming Qi, Jiang Zhou

**Affiliations:** 1 Institute of Forest Ecology Environment and Nature Conservation, Chinese Academy of Forestry, Beijing, 100091 China; 2 Department of Anthropology, Program in Ecology, Evolution, and Conservation Biology, University of Illinois, Urbana, IL USA; 3 Bawangling Branch of Hainan Tropical Rainforest National Park Administration, Changjiang, Hainan China; 4 School of Karst Sciences, Guizhou Normal University, Guiyang, Guizhou China

**Keywords:** *Nomascus hainanus*, Social structure, Mating system variability, Extinction risk

## Abstract

Understanding flexibility in the social structure and mating strategies of the world’s last remaining population (35 individuals) of wild Hainan gibbons (*Nomascus hainanus*) is critical for developing effective management plans to aid in their population recovery. Three of the five remaining Hainan gibbon groups (A, B, and C) currently live in a social unit characterized by two or three adult males, two reproducing adult females, and offspring. A fourth group (D) contains one adult male, two adult females, and offspring, and Group E contains a single adult male–adult female pair with a young infant. In this study, we describe observations of copulations between multiple resident males and one of the two resident females in Group C. Group C is best described as a small multi-male/multi-female group. We found that this breeding female (F2) solicited copulations from two resident adult males (M1 and M2) on the same day, and also mated with each of these two males on different days. Resident males were not observed to interrupt the mating pair. Although factors such as a biased adult sex ratio, severe population disruption, and habitat degradation can help explain variation in group composition and mating strategies in Hainan gibbons, we argue that there exists considerable mating system variability across gibbon species, and that this variability offers important insights into male and female Hainan gibbon group structure and reproductive strategies.

**Supplementary Information:**

The online version contains supplementary material available at 10.1007/s10329-022-00983-5.

## Introduction

**Fig. 1:**
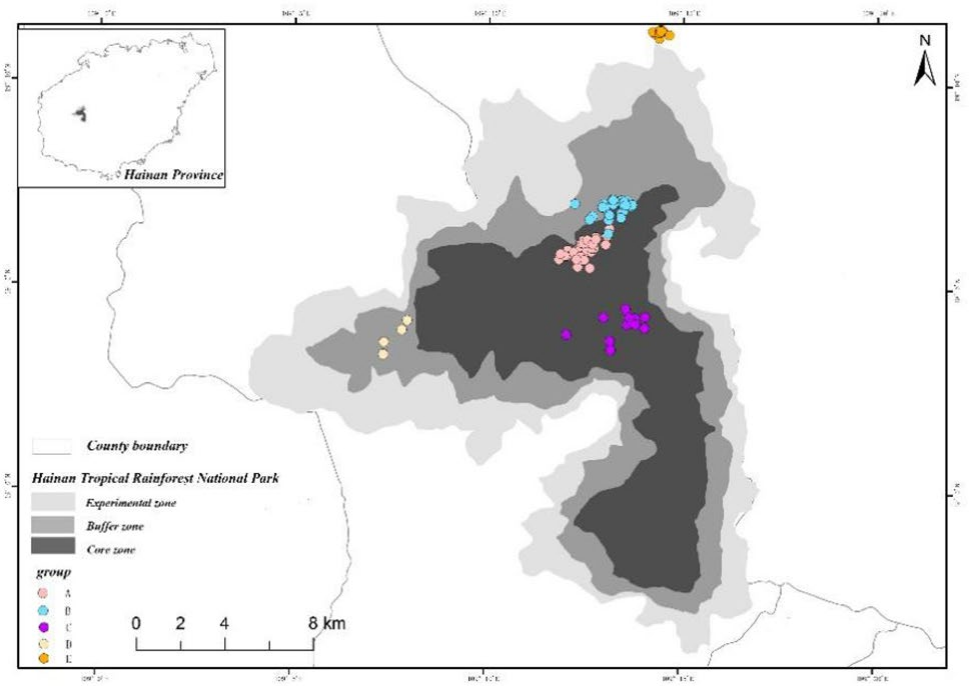
Distribution of the five remaining groups of Hainan gibbons

**Fig. 2:**
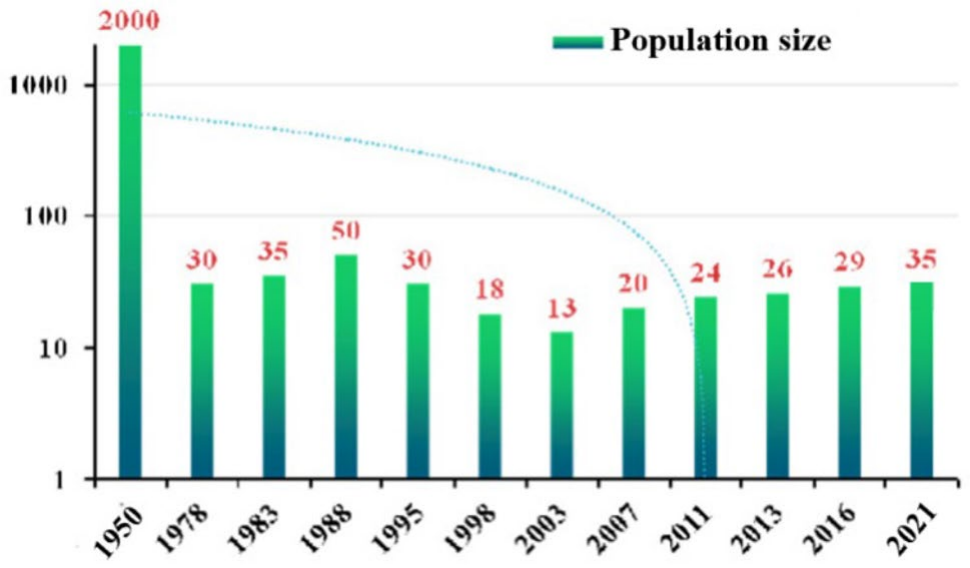
Dynamic changes in the population size of Hainan gibbons over the past 70 years

**Fig. 3:**
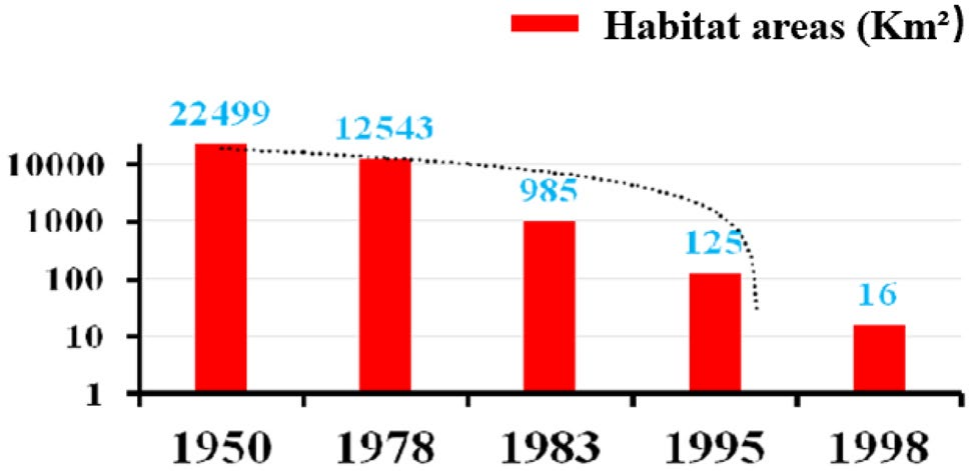
Dynamic changes in the area of remaining suitable habitat for Hainan gibbons

The Hainan gibbon is the world’s most endangered species of ape. At present, only 35 Hainan gibbons exist in the wild, including 30 individuals residing in five social groups plus five floater or extra-group individuals (Hainan Tropical Rainforest National Park Press Conference 2021; Table 1). This species is endemic to China and the entire remaining population inhabits an area of some 16 km^2^ located in the Bawangling sector of the Hainan Tropical Rainforest National Park (HTRNP), Hainan Island, China (Fig. 1). Beginning in the 1960s and continuing for 40 years, Hainan island was heavily logged for industrial agriculture and development, reducing the habitat available to these small-bodied apes by 99% and the population size by 95% (Guo et al. 2021; Zhang et al. 2010) (Figs. 2, 3). More recently, Hainan gibbons have been elevated to first-class key protected wildlife status in China, and are listed as Critically Endangered by both the International Union for Conservation of Nature (IUCN, 2021) and China’s Red List (Jiang et al. 2016). In 2019, the Chinese government created the HTRNP to protect this last remaining population of Hainan gibbons.

**Table 1:** The size and composition of Hainan gibbon groups as of August 2021

Group	Adult male	Adult female	Immatures	Group size	Notes
Group A	2	2	2	6	One of the adult males was born into this group
Group B	2	2	4	8	One of the adult males was born into this group and the other immigrated into the group. A new infant was born into this group in January 2021
Group C	3	2	3	8	One of the adult males was born into this group
Group D	1	2	2	5	This group formed in 2015, and initially was composed of one adult male and one adult female. A second adult female joined the group in 2018. In 2019, the original female died, and a new adult female joined the group that same year. A new infant was born into this group in January 2021
Group E	1	1	1	3	This group originated in 2019 and its home range borders, but lies outside, the protected reserve
Solitary gibbons	2	2	1	5	Two adult females, two adult males, and one juvenile have been identified
Total	11	11	13	35

Gibbons (primate family Hylobatidae) represent a taxonomically diverse radiation of small-bodied Asian apes that includes four genera (*Hoolock, Hylobates, Nomascus, Symphalangus*) and 18 species (IUCN 2021). Five species are listed by the IUCN as Critically Endangered, 12 species are Endangered, and one species, *Hoolock leuconedys*, is considered Vulnerable (IUCN 2021). Although gibbons were long thought to form pair-bonded family groups and exhibit a monogamous mating pattern, several species of crested gibbons *(Nomascus concolor, Nomascus nasutus, Nomascus gabriellae,* and *Nomascus hainanus*) are reported to be polygamous and form stable groups composed of one adult male and two adult females (Hu et al. 2018; Kenyon et al. 2011; Thanh 2011; Zhou 2008). Both resident females are reproductively active, and in several cases represent adult mother-adult daughter pairs. Groups with multiple adult males and more than two adult females also have been reported (Hu et al. 2018).

New gibbon groups can form in a variety of ways. In some instances, maturing subadults or young adults that have dispersed from their natal group join a lone opposite-sex adult. In other cases, a floater adult male or adult female may enter an established group and displace a current same-sex resident, or enter an established group after the death of a same-sex resident (Reichard 2009). In contrast, little is known concerning the mating strategies and group formation of Hainan gibbons. In this species, individuals reach sexual maturity at ca. 5–8 years of age, and during this period exhibit ontogenetic changes in body coloration associated with sexual dichromatism. Subadults of both sexes are black. At sexual maturity, males remain black whereas females become predominantly yellow or buff-colored, except for a black crest of fur on the top of their head. Female Hainan gibbons give birth every 2–3 years (Zhou et al. 2008) and are reported to solicit copulations by presenting their hindquarters to an adult male, rapidly shaking their entire body, and engaging in what has been described as a robot-like dance (Zhou 2008). Additional data on the mating and residence strategies of Hainan gibbons would greatly assist conservation efforts in assessing the effects of demographic changes in group size, composition, and patterns of sex-based dispersal on the population persistence and conservation status of this Critically Endangered species.

Here we provide data on the size, age/sex composition, and history of all five remaining wild groups of Hainan gibbons. In addition, we present detailed observations on mating patterns in Group C. Group C contains eight members including three adult males (M1, M2, and M3), two adult females (F1 and F2), one juvenile (4 years old), and two infants (one infant is 14 months old and the other is 24 months old). These infants are the offspring of adult females F1 and F2, both of whom are active breeders in the group. In presenting these data, our primary goal is to examine the degree to which patterns of mating and group composition in this heavily impacted primate population resemble patterns of group composition and mating in other gibbon taxa.

## Methods

We studied the behavior and social composition of gibbon Group C from September 2011 to August 2021 during five consecutive days per month. On average, the gibbons were followed for 9 h per day and data on group location, activity budget, and social interactions were collected using a 5 min scan sampling method at 15 min intervals (Altmann 1974). Individuals were identified based on morphological characteristics and patterns and changes in hair color. Detailed ad libitum data were collected on mating behavior. All observations involved the use of a Kowa TSN-883 telescope.

In 2011, Group C was composed of three resident adults: M1, F1, and F2. In 2013, M2 joined the group. Adult group membership remained stable (two adult males and two adult females) for the next 6 years. In 2019, M3, who is the son of M1 (paternity was determined genetically, M3 was born into the group in 2013), attained full sexual maturity, and he has remained a member of his natal group.

## Results

### Group size, composition, and history

As of August 2021, four of the five established Hainan gibbon groups each contained two breeding females, and three of the groups contained two or three adult males (Table 1). Group E, which formed in 2019, is the smallest group and contains one adult male, one adult female, and one infant. Group A and Group B formed in 1970 (Zhou 2008). Group C formed in 2011, and Group D formed in 2015. The mean group size was 6 ± 2.2 individuals, and the adult male-to-female sex ratio in established groups was 1:1.2 (Table 1). We note that in Groups A, B, and C, one of the two resident adult male group members was a natal male. We also documented two cases in Group D of an adult female successfully immigrating into the group and breeding, and one case (Group C) of a non-natal adult male successfully joining an established group that contained one resident adult male (Table 1).

### Mating and behavioral observations in Group C

All three adult males in Group C engaged in traditional morning calls. Typically, M1 initiated the morning chorus and was joined after a few minutes by M2. During these morning choruses, M1 and M2 were located within a few meters of each other. Once the morning chorus ended, M3, who was generally positioned tens of meters from the other adult males, would sing on his own. On most mornings, females F1 and F2 responded to M1s vocalizations and joined him in singing. These females, however, did not sing when only M2 or M3 vocalized. Between March 2019 and April 2021, we opportunistically observed five instances of mating in Group C. On March 21, 2019, we observed F2 to solicit matings with both M1 and M2. On July 11, 2020, we observed F2 to solicit and copulate twice with M2, and on April 25, 2021, we observed F2 to engage in an unsolicited copulation with M1. We detail our observations below:

#### March 21, 2019

At 11:40 on March 21, 2019, F2, who was pregnant (F2 gave birth 2 months later, in May 2019; gestation in gibbons is approximately 7 months), engaged in a robot-like ritual mating solicitation dance that lasted 1 min. M1 then approached F2 and attempted to mate. The copulation lasted for only a second, the male did not engage in thrusting behavior, and this mating appeared to be unsuccessful. After M1 moved away from F2, she was approached by M2, who attempted to mate with her. This mating attempt also did not result in ejaculation.

#### July 11, 2020

At 9:00 am and at 11:30 am on July 11th, we observed M1 aggressively chasing M2. At 12:00 noon, F2 solicited M2 by engaging in a robot-like dance movement and a rapid shaking of her entire body. M2 responded by approaching F2, who grabbed the male’s foot, groomed him, and presented to him. M2 reacted by jumping onto another tree. F2 continued to solicit M2, and at 12:11, M2 joined F2 (Supplementary Fig. 1) and they successfully copulated. Following this first mating, the male squatted and cautiously scanned the canopy, and at 12:12, the pair engaged in a second copulation. During the mating process, neither F2 nor M2 vocalized. Later that same day, F2 mated with M1. During each of these mating events, F2 held her 13-month-old infant. Given that Hainan gibbon females may give birth every 2 years, it is possible that F2 was ovulating at the time of these copulations.

#### April 25, 2021

On April 25th, 2021 at 11:05, F2 was holding her now 23-month-old infant. M1 approached F2 and they copulated. After mating, M1 remained within 0.5 m of F2. F2 then engaged in a robot-like ritual mating solicitation and moved closer to M1. However, they did not copulate.

## Discussion

Hainan gibbons are among the world’s most endangered primate species. Only 35 gibbons, distributed across five social groups plus five lone or floating individuals, remain in the wild. Here we present data on group size, composition, and history, and describe five observed matings in Group C. This group contained eight individuals including two adult females and three adult males. The adult composition of this group has remained fairly stable since 2013. We observed two copulations between adult female F2 and adult male M1, and three copulations between F2 and adult male M2. The third adult male, M3, who was a natal male, was not observed to copulate. We use this information, along with data on group size and composition, to better understand gibbon grouping and mating variability.

Several primate researchers have highlighted an important distinction between the social system (the number, age, sex, and behavioral interactions of individuals who share a common home range, and travel, feed, forage, and rest together over the course of months or years), the mating system (the set of individuals who copulate), and the breeding system (the set of individuals who contribute genes to the next generation) (Garber et al. 2016; Kappeler Peter et al. 2002; Reichard 2009; Garber et al. 2016). In species that are characterized by a single adult male and a single adult female, and in species that form a single-male/multi-female group, it is often assumed that the same set of individuals that comprise the mating and social system, also comprise the breeding system. However, several recent studies have shown that primate mating and breeding systems can be highly flexible, and “transitions between pair-living and polyandrous grouping, as well as monogamous and polygamous mating strategies, can occur in response to environmental and social changes or because the use of multiple diverse mating strategies maximizes the reproductive success of group members” (Reichard 2009: 348). For example, a recent study by Qi et al. (2020) found that although the basic social unit of golden snub-nosed monkeys (*Rhinopithecus roxellana*) is composed of a single adult male and 3–5 adult females, more than 50% of offspring born into these one-male units were sired by extra-group males and not the group’s leader male. In the case of the forked-mark lemur (*Phaner furcifer*), despite residing as a single male–female pair, extra-pair paternity was documented in six of seven offspring born over a 4-year period (Schulke et al. 2004). And even in the case of gibbon species that are reported to form long-term pair bonds, such as white-handed gibbons (*Hylobates lar*) and siamangs (*Symphalangus syndactylus*), extra-pair copulations have been observed (Palombit 1994; Reichard 1995). For example, Reichard (1995) reported that in *H. lar*, 12% of observed copulations involved extra-group males, with the majority of these copulations occurring during a female’s fertile period. Based on paternity analysis, Barelli et al. (2013) confirmed that 3 of 41 white-handed gibbon infants were sired by extra-group males. Similarly, in polygamous gibbon groups, not only does the resident male mate with both of the groups’ resident females, but resident females also have been observed to engage in extra-pair copulations. In the case of the yellow-cheeked gibbon (*N. gabriellae)*, DNA evidence indicated extra-pair paternity in 1 of 10 of study groups (Kenyon et al. 2011). In that study, this male sire was identified as a lone or floating male, who was not part of an established group. Extra-pair copulations between male and female residents from neighboring groups (Huang et al. 2013) and resident females and floater males also have been reported in black crested gibbons (*N. concolor*; Huang et al. submitted). Not only can floaters play an important role in increasing gene flow and genetic diversity, in species in which the number of breeding adults per group is small, the presence of floaters offers a mechanism for the rapid replacement of lost breeders, thereby ensuring group continuity (Robles et al. 2017).

Moreover, within-species variation in mating and reproductive strategies can influence variation in extinction risk, especially among small and endangered populations (Lootvoet et al. 2015). In this regard, Leach et al (2020) examined the effects of stochasticity or random events on extinction probability in simulated social groups that differed in size and systems of mating. These authors found that in general, taxa characterized by smaller group size and a monogamous mating system had a higher expected risk of extinction. This is supported by a study by Lootvoet et al. (2015) indicating that species characterized by greater mating flexibility exhibited a reduced risk of extinction. It appears that species that adhere to a strictly monogamous mating pattern experience problems of new group formation and established group stability when the adult sex ratio differs significantly from 1:1 (Leach et al. 2020).

Ecological factors related to diet, nutrition, and foraging efficiency also can affect species fertility and survivorship (Lambert 2011). It is hypothesized that highly frugivorous species, such as gibbons, and species that exploit large home ranges (i.e. require a large search area to find food) are more vulnerable to stochastic process-associated reduction in food availability and local extinction (Lootvoet et al. 2015). These results have important implications for developing effective species conservation plans, especially in cases in which considerations include translocation of individuals of particular age/sex classes across subpopulations, introducing captive individuals back into the wild, creating corridors that reunite previously isolated subpopulations or groups, and creating protected areas that contain suitable native habitat (Lootvoet et al. 2015).

Given that almost 99% of the habitat of Hainan gibbons has been lost over the past 50 years, and the population was reduced to only 12 individuals in 2002, the degree to which the social organization, mating system, and breeding system of the five extant groups closely approximates the modal pattern for this species remains unclear. However, the fact that two of the five groups are characterized by a two-male/two-female group structure, one group by a three-male/two-female group structure, one group by a one-male/one-female group structure, and one group by a one-male/two-female group structure, each of which has been reported in other crested gibbon species (Fan et al. 2021; Hu et al. 2018) (Table 2), suggests that the observed variability may represent the range of group structures traditionally present in Hainan gibbons.

**Table 2:** Observed variability in crested gibbon social, mating, and breeding systems^1,2,3^

Species	Group structure	Extra-group mating	Mating initiator	Sexual maturity	Floaters	Emigration
*N. annamensis* (Endangered)	1 M + 1 F; 1 M + 2 F	None	M; F	5–8	None	None
*N. concolor* (Critically Endangered)	1 M + 1 F; 1 M + 2 F; 2 M + 2 F	+	F	8–12	+	M; F
*N. gabriellae* (Endangered)	1 M + 1 F	+	UNK	6–7	+	M; F
*N. hainanus* (Critically Endangered)	1 M + 1 F; 1 M + 2 F; 2 M + 2 F	None	F	5–8	+	M; F
*N. leucogenys*^*3*^ (Critically Endangered)	1 M + 1 F	None	UNK	7–8	None	None
*N. nasutus* (Critically Endangered)	1 M + 2 F	+	UNK	6–7	None	None
*N. siki* (Critically Endangered)	1 M + 1 F	none	UNK	4–8	None	None

^1^- Data on conservation status of *Nomascus* species are from the IUCN Red List consulted November 11, 2021

^2^- Data on *Nomascus* social, mating, and breeding systems are from Rowe and Myers (2006) All the World’s Primates and Huang et al. 2013

^3^- Data on *Nomascus leucogenys* (Primates: Hylobatidae) Author: Lee E. Harding. Mammalian Species, Vol. 44, No. 1 (Jan., 2012), pp. 1–15

Note: “*M*” represents male, “*F*” represents female, “+” represents existence, “*UNK*” indicates information is unknown

At present, however, we have no observations of resident females mating with a floater male or resident males mating with a floater female in Hainan gibbons. Mating between resident females and neighboring males in established groups and mating between resident females and floating males have been documented in other crested gibbon species (Huang et al. 2013; Huang et al. submitted; Kenyon et al. 2011). At present, the adult male-to-female sex ratio in established groups of Hainan gibbons is 1:1.2 (Table 1). There also are five floater gibbons in our population. Two of these floaters are adult females, two are adult males, and one appears to be an immature. Fan et al. (2021) report that in captive gibbons, the sex ratio at birth does not deviate significantly from 1:1. However, in one wild population of *Nomascus concolor,* the sex of 7 of 16 infants were unambiguously determined, and six were male and one was a female (Huang et al. 2013).

Given the relatively slow reproductive rate (interbirth interval [IBI] = 24–42 months) and late age at first reproduction (5–10 years of age) in *Nomascus* gibbons (Fan et al. 2021), short-term perturbations in the sex ratio at birth, especially in small populations, could have a significant effect on group size, composition, and male and female reproductive strategies. However, despite experiencing severe habitat degradation and near population collapse, the world’s last remaining five groups of Hainan gibbons appear to exhibit a group size, composition, and patterns of mating consistent with other species of *Nomascus* gibbons (Table 2). The ability of these critically endangered primates to form stable social and breeding groups, similar in composition to other gibbon species despite their extremely small population size and severely impacted habitat, offers hope for their population recovery. Overall, it appears that certain aspects of crested gibbon social and reproductive behavior are highly conservative and present in several species (i.e. small group size, tolerance for a small number of same-sex adults, male dawn chorus, male–female dueting, female sexual solicitation of male partners). In contrast, other aspects of their social and reproductive behavior (i.e. the degree to which females solicit matings from resident males, neighboring males, or floating males; mating partner fidelity; whether groups contain one, two, or three males; and whether resident breeding females are mother-daughter pairs or unrelated) appear to be more variable and dependent on local ecological and demographic conditions (see Garber et al. 2019 for a similar discussion of trait variability and trait stability in the social and reproductive behavior of common marmosets).

Finally, given the relatively few field studies on Hainan gibbons, our knowledge of their behavior, diet, ecology, and reproductive strategies remains limited. This situation is exacerbated by the fact that all Hainan gibbons live in a highly altered landscape, making conservation planning and effective population management extremely difficult. For example, whereas most gibbon species exploit home ranges of between 30 and 540 ha, the home range of Hainan gibbons is reported to be between 548 and 987 ha (Deng and Zhou 2018). The large range of Hainan gibbon groups is likely the result of severe habitat fragmentation and the conversion of their natural lowland habitat (below 700 m) to agricultural plantations forcing individuals to forage and feed at altitudes of between 700 and 1280 m (Deng and Zhou 2018). It remains unclear whether dietary resources in these disturbed upland forests provide a sufficiently high-quality diet, enabling the Hainan gibbons to expand their population over time. Moreover, the availability of suitable habitat is likely to affect the number and viability of floaters (Robles et al. 2017) in the population. In gibbons, floaters may play a critical role in avoiding group disintegration by rapidly replacing lost breeders. Thus, detailed and long-term data on the process of group formation, patterns of dispersal, group stability, the role of floaters in expanding genetic diversity, and information on nutritional ecology and diet are essential for developing effective management plans to protect and expand the area of suitable habitat for this primate species. If Hainan gibbons are to be saved from extinction, population recovery will require the Chinese government to prioritize programs that restore Hainan Island’s natural forests, fund long-term research, and protect the world’s last remaining population of Hainan gibbons.

## Supplementary Information

Below is the link to the electronic supplementary material.

Supplementary file1 (DOCX 133 KB)
