# Supplementary material for: Diverse grouping and mating strategies in the Critically Endangered Hainan gibbon (Nomascus hainanus)
Source: Primates. 2022 Mar 24;63(3):237–43. doi: 10.1007/s10329-022-00983-5 (PMC9061651; doi:10.1007/s10329-022-00983-5)
Supplement: Supplementary file 1 — Supplementary file1 (DOCX 133 KB) [file 10329_2022_983_MOESM1_ESM.docx]

**Supplementary：**


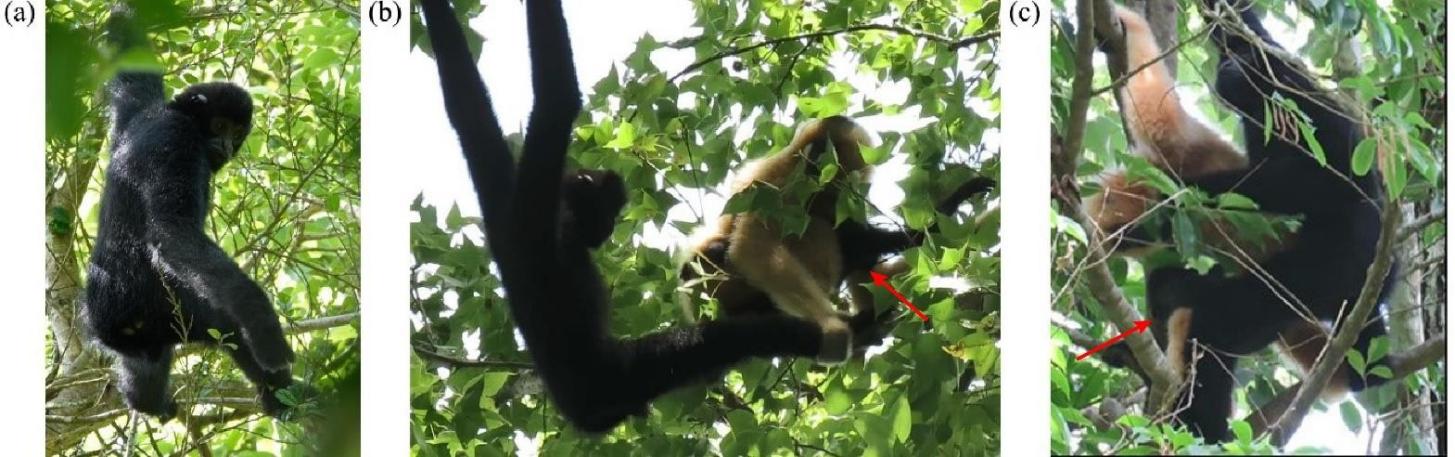


Fig 4 (1a) The distinctive ear notch of M2; (1b) F2 soliciting M2: (1c) F2 and M2 mating (notice that F2 was holding her 13 month old infant (red arrow) during the mating process). Photos taken on July 11, 2020 258
